# Association of recurrent common infections and subclinical cardiovascular disease in Mexican women

**DOI:** 10.1371/journal.pone.0246047

**Published:** 2021-01-26

**Authors:** Priscilla Espinosa-Tamez, Martin Lajous, Carlos Cantú-Brito, Ruy Lopez-Ridaura, Adriana Monge, Elsa Yunes, Beatriz L. Rodríguez, Luis Espinosa, José Sifuentes-Osornio, Andres Catzin-Kuhlmann

**Affiliations:** 1 Center for Population Health Research, National Institute of Public Health, Tlalpan, Mexico City, Mexico; 2 Department of Global Health and Population, Harvard T.H. Chan School of Public Health, Boston, MA, United States of America; 3 Division of Neurology and Psychiatry, National Institute of Medical Sciences and Nutrition Salvador Zubirán, Tlalpan, Mexico City, Mexico; 4 Escuela de Medicina y Ciencias de la Salud, Tecnologico de Monterrey, Monterrey, Nuevo Leon, Mexico; 5 Department of Geriatric Medicine, University of Hawaii, Honolulu, HI, United States of America; 6 Department of Medicine, National Institute of Medical Sciences and Nutrition Salvador Zubirán, Tlalpan, Mexico City, Mexico; Columbia University Medical Center, UNITED STATES

## Abstract

**Background:**

Acute and agent-specific chronic infections have been associated with increased cardiovascular risk, however data on the burden of common recurrent infections on cardiovascular disease is limited. We hypothesized women with greater exposure to uncomplicated common infectious events had an increased risk of subclinical cardiovascular disease (sCVD).

**Methods:**

In a cross-sectional study, we assessed the relation of recurrent infections and carotid artery intima-media thickness (IMT) in 1946 disease-free women from the Mexican Teachers’ Cohort. Through 2012–2016, participants answered structured questions on respiratory, urinary and vaginal infections during the previous year and their IMT was measured using ultrasound by standardized neurologists. We defined sCVD as mean right and left IMT ≥0.8 mm or the presence of atheromatous plaque. Multivariable linear and logistic regression analyses were used to evaluate the association of infectious events with IMT and sCVD adjusting for age, sociodemographic, and cardiovascular risk factors.

**Results:**

Among participants (50±5 years) 13% reported no infections, 20% one infection and 67% three or more episodes. Overall prevalence of sCVD was 12%(n = 240). Adjusted models for logistic regression showed that women with 2 or more infections had 91% higher odds of sCVD (OR 1.91; 95%CI 1.16, 3.13) compared to women without infections (p-trend:0.015). Sub-analyses by type of infection resulted not significant. Linear regression analysis did not show a significant association between mean IMT and recurrent infections.

**Conclusions:**

Recurrent infectious events in young adult women are associated with greater sCVD, which supports the hypothesis of low-grade chronic inflammation in the pathophysiology of cardiovascular disease.

## Introduction

Inflammation is associated with atherosclerosis, which increases the risk of cardiovascular events [1]. Factors and mechanisms involved in inflammation and infection are also involved in the pathogenesis of cardiovascular disease: macrophages are found in atherosclerotic plaques and inflammatory markers like high-sensitivity C-reactive protein (CRP) levels can predict coronary events [2]. Infections have been determined to increase the risk of cardiovascular diseases, including acute myocardial infarction, ischemic stroke and atherosclerosis [3–5].

Several studies have assessed the relation between agent-specific infections and cardiovascular disease [2, 6–9]. *Chlamydia pneumoniae* and Herpes simplex virus type 1 have been associated with coronary heart disease risk, especially in subjects with increased levels of CRP [2]. Respiratory [10], cytomegalovirus [11], tuberculosis [12], and influenza infections [13–15], have been associated with acute and long-term risk of myocardial infarction [16]. Higher risk of ischemic stroke [10, 17, 18] has been found in patients with tuberculosis [19], influenza [20], respiratory [10], cytomegalovirus and herpesvirus simplex [21, 22] infections.

Infections cause atherosclerosis by direct (infecting vascular cells and triggering immune response), or indirect mechanisms (circulating inflammatory factors) [6]. Several agent-specific infections have been found to cause atherosclerosis through both mechanisms, including cytomegalovirus [23–25], herpes simplex virus [26–28], influenza [29], Epstein-Barr virus [30], hepatitis virus [31, 32], human immunodeficiency virus [33] and human papillomavirus [34].

However, low-grade chronic inflammation, which would be expected to exist in individuals with persistent or recurrent infections, could also be involved in a greater predisposition for atherosclerotic disease. “Infectious burden”, the combined and aggregated activity of several infections was found to be significantly associated with maximum carotid plaque thickness [35–38]. Chronic obstructive pulmonary disease exacerbations, as well as documented urinary tract infections and self-reported periodontitis were also related to an increased risk of atherosclerosis defined by intima-media thickness, even without other vascular risk factors [39].

The “pathogen burden” theory proposes that the element that should be considered relevant in the progression of atherosclerosis is the total number of infectious pathogens in the lifetime of an individual [40, 41]. It has been shown that infections of different organ systems are associated with increased cardiovascular risk, which suggests that chronic inflammation itself could have a generic role, rather than the specific infection site [4, 42]. Despite the evidence of both acute and agent-specific chronic infections’ association with increased cardiovascular risk, data on the burden of common recurrent infections and cardiovascular disease is extremely scarce and in the Mexican population it is nonexistent. Therefore, we hypothesized women with greater exposure to uncomplicated common infectious events had an increased risk of sCVD, which is defined by the presence of atherosclerosis of carotid arteries by high-resolution ultrasound imaging.

## Material and methods

### Study population

The Mexican Teachers’ Cohort (MTC) is a large prospective cohort study of 115,314 female teachers aged 25 years and older, followed since 2006 and 2008 across 12 Mexican states. At baseline, the participants responded to a self-administrated questionnaire on demographic, reproductive, lifestyle, diet, and health status characteristics [43]. Between 2012 and 2016, 3,613 study participants aged 40 years and older and living within a 50 km radius from clinical sites in three states (Chiapas, Yucatán, and Nuevo León) were invited to participate in a cardiovascular disease ancillary study. There were 2,390 women who volunteered (65.6%).

In this cross-sectional study, we excluded teachers that had a history of myocardial infarction or cerebrovascular disease (n = 17), those who reported implausible amounts of infections during the previous year (≥60 site specific infections, n = 4), women without carotid IMT measurements (n = 272), and those who did not answer the infections section of the questionnaire (n = 151). Thus, our final analyses included 1,946 women. The study was approved by the Institutional Review Board at the National Institute of Public Health, and at the Escuela de Medicina, Tecnologico de Monterrey. All participants provided informed consent.

### Assessment of infections

During the clinical evaluations, participants responded to: “How many respiratory infections (sore throat, cold, flu, sinusitis) have you suffered during the previous year?”, “How many urinary infections (burning when urinating, bladder pain, urgency to urinate) that required antibiotic treatment…”, and “How many vaginal infections (vaginal burning, itching, abnormal discharge)…”. Participants wrote down a number for each of these three questions. Since no standard validated questionnaire existed for the evaluation of common recurrent infections, we designed and reviewed this questionnaire with practicing physicians of different specialties. Due to the nature of the clinical subcohort, and the difficulties to access the population previous to the clinical evaluations, a pre-test was not carried out.

### Carotid artery intima-media thickness measurement

Carotid arteries were examined with a lineal array transducer operating at a frequency of 10 MHz using SonoSite MicroMaxx ultrasound scanning and an Asus laptop with M’AthStd Software (Intelligence in Medical Technologies). Reproducibility of the IMT measurement was evaluated in Chiapas and Yucatán. Reproducibility was r = 0.89 (95% CI: 0.85, 0.93) for Chiapas and r = 0.92 (95% CI: 0.85, 0.93) for Yucatán [44]. Examination included visualization of external, internal, and common carotid arteries with patients in a supine position with their head rotated 20° to 30°. IMT was measured along a 10 mm length starting 5 mm below the far wall end of the common carotid artery where the carotid bifurcation was clearly visible. We obtained the mean IMT for the 10 mm segment of each common carotid arteries to calculate the overall mean. Using the measurement from the adventitia-media interface up to the intima-lumen interface, plaque was defined according to the Mannheim consensus: focal structures of at least 0.5 mm encroachment into the lumen, 50% greater thickness compared to the surrounding IMT, or a thickness greater than 1.5 mm [45]. IMT was measured by standardized neurologists, and sCVD was defined as mean right and left IMT ≥0.8 mm or plaque appearance.

### Covariates

Since 2008 when the baseline questionnaire was recovered, teachers answered follow-up questionnaires in 2011, and during clinical evaluations complementary ones. These assessed socioeconomic status, age at diagnosis and treatment of chronic diseases, menopausal status, physical activity, smoking, and alcohol consumption.

During the clinical evaluations in 2012–2016, teachers fasted for at least 8 hours; this was corroborated with the participants by clinical personnel. The physical examination included anthropometric measurements (weight, height, waist and hip circumferences), blood pressure, carotid ultrasound and ankle-brachial index determination. Previously standardized clinical technicians placed blood pressure cuffs on the 4 extremities, and blood pressure measurements were performed automatically (VaSera VS-1000; Fukuda Denshi). Standardized personnel performed weight and height measurements with the use of an electronic digital scale (Tanita Corp, Arlington Heights, Illinois, USA) to the nearest 0.1 kg and a wall stadiometer (Seca Corp; Hamburg, Germany) to the nearest millimeter. Blood samples were obtained after 8-hour fasting by venipuncture by trained nurses and were processed within 30 minutes. Glucose, low-density lipoprotein cholesterol (LDL), and high-density lipoprotein cholesterol (HDL) were assayed using standard methods.

Socioeconomic status was established as tertiles depending on the amount of seven household assets: computer, vacuum cleaner, microwave oven, cell phone, phone, car, and internet. Body mass index (BMI) was calculated as weight in kilograms divided by the square of height in meters, and obesity was defined as a BMI ≥ 30 kg/m^2^. Diabetes, hypertension, and hypercholesterolemia were either self-reported in 2008 and 2011 questionnaires (diagnosis or treatment) or diagnosed at clinical evaluation as follows. Diabetes: fasting glucose >125 mg/dL; hypertension: systolic blood pressure ≥ 140 mm Hg or diastolic blood pressure ≥ 90 mm Hg; and hypercholesterolemia: total cholesterol ≥ 240 mg/dl or LDL cholesterol ≥ 160 mg/dl. Uncontrolled diabetes was defined at the clinical evaluation as fasting glucose ≥200 mg/dL.

### Statistical analysis

For carotid IMT continuous analysis measurements were log-transformed, and back-transformed for interpretation. Respiratory, urinary tract, vaginal and total infections were categorized as “0”, “1” and “2 or more” infectious episodes in the previous year.

Multivariable linear and logistic regression analyses were used to evaluate the association of infectious events with IMT and sCVD, respectively, comparing the higher infectious events category to the “0 infections” category for each type of infection and for total infectious events. Multivariable models were obtained adjusting for covariates: Model 1 adjusted for age and, to account for geographical distribution of participants, for evaluation site; Model 2 (“environmental factors”) added socioeconomic status, education level, smoking status, and alcohol consumption; and Model 3 (“comorbidities”) added diabetes, hypertension, hypercholesterolemia, menopausal status, and body mass index for adjustment. Logistic regression estimates and confidence intervals are described in the format: (odds ratio [OR]; 95% confidence interval [CI]). Linear trend (p-trend) was estimated including the median value of infections of each category of total infectious events as a continuous variable in the models. Since we only evaluated preestablished complementary comparisons, no correction for multiple comparisons was performed. Stratified analyses were conducted to evaluate effect modification using median BMI (28.5 kg/m^2^) and median age (49 years) of the study population. This was evaluated by including cross-product terms of the median value of infections of each category of total infectious events as a continuous variable and categories of age and BMI (p-interaction).

Since there is no standard classification for exposure to infectious events, we performed sensitivity analyses with different categorizations of total infectious events: (1) with total infections categorized as: “0”, “1”, “2” and “3 or more”; and (2) total infections categorized considering a hypothetical yearly distribution as follows: “0”, “1 event during the year”, “1 infection per semester” (reported as 2), “More than one per semester” (≥3 and <12 events), and “1 event per month or more” (≥12 reported).

We also performed a sensitivity analysis (3) with subclinical cardiovascular disease defined as right or left IMT ≥0.8 mm or plaque.

Statistical analyses were performed using SAS version 9.4 (SAS Institute, Cary, NC).

## Results

The mean age ±SD of the 1946 women included was 50 ±5 years. Among participants, 22% reported no respiratory infections during the previous year, 30.1% one infection and 47.9% two or more. Regarding urinary and vaginal infections, the distributions were 64.9%, 20.7%, and 14.4%; and 64.3%, 18.7%, and 17% of participants, respectively. Considering total infections (respiratory, urinary, and vaginal combined) 12.7% reported none, 20% one, and 67.3% two or more. Age-adjusted sociodemographic and cardiovascular risk factors of the population are shown in Table 1, according to the number of total infections in the previous year.

**Table 1:** Characteristics of 1946 women from the Mexican Teachers’ Cohort (MTC) by categories of total infectious events during the last year, age adjusted.

	Categories of total infectious events		
	0	1	2 or more
	n = 246 (%^a^)	n = 390 (%^a^)	n = 1310 (%^a^)
Age (SD), y^b^	50.9(5.4)	50.6(5.1)	49.2(5.1)
Educational Level			
• Highschool or less	63 (24.7)	114 (29.7)	382 (29.2)
• Undergraduate	134 (55.6)	206 (53.8)	718 (54.4)
• Graduate	49 (19.7)	70 (16.4)	210 (16.4)
Socioeconomic Status			
• Tertile 1	73 (31.2)	103 (28.5)	464 (34.8)
• Tertile 2	41 (16.7)	55 (14.5)	231 (17.4)
• Tertile 3	132 (52.1)	232 (57)	615 (47.8)
Smoking			
• Nonsmokers	194 (80.2)	305 (79.3)	1036 (78.7)
• Ex-smokers	27 (10.5)	55 (13.3)	177 (13.8)
• Current smokers	24 (8.9)	29 (7.2)	90 (6.9)
Alcohol intake (SD), servings/week	0.6(1.1)	0.5(1.8)	0.5(0.9)
BMI (SD), kg/m^2^ ^c^	29.3(5.7)	29.5(5.4)	29.4(5.5)
Obese	93 (36.7)	148 (37)	501 (38.7)
Diabetes	24 (9)	26 (6.4)	92 (7.2)
Uncontrolled diabetes (fasting glucose>200)	6 (2.4)	5 (1.3)	29 (2.3)
Hypertension	28 (10.5)	47 (11.5)	185 (14.5)
Hypercholesterolemia	95 (35.8)	115 (29.1)	418 (32.5)
Menopausal Status			
• Premenopausal	95 (45.2)	141 (41.7)	591 (42.3)
• Postmenopausal	132 (46.9)	201 (45.5)	545 (44.9)
• Unknown	19 (7.9)	48 (12.8)	174 (12.8)

^a^ Values in parenthesis are percentages unless otherwise specified. Percentages were age-standardized to the age distribution of the study population.

SD = standard deviation.

^b^ Value is not age adjusted.

^c^ Three participants had a missing BMI because they did not have complete weight or height information.

Higher educational level was related to a lower number of infections during the previous year. Women in the highest category of total infectious events were more likely to have hypertension (Table 1), greater IMT measurements and sCVD (Table 2). Among participants (n = 1,946) the overall prevalence of sCVD was 12.3% (n = 240).

**Table 2:** Carotid findings in 1946 women from the MTC by categories of total infectious events during the last year, age adjusted.

	Categories of total infectious events		
	0	1	2 or more
	n = 246	n = 390	n = 1310
Right Mean IMT(SD), mm	0.672(0.116)	0.670(0.098)	0.680(0.110)
Left Mean IMT(SD), mm	0.686(0.094)	0.697(0.108)	0.701(0.115)
Mean IMT(SD), mm	0.679(0.091)	0.683(0.088)	0.691(0.099)
Plaque, %	1.5	2.9	3.0
SCVD, %	8.8	11.3	13.5

Values were age-standardized to the age distribution of the study population.

SD = standard deviation.

Although not statistically significant, a trend could be appreciated in the relation between amount of respiratory, urinary, and total infections during the previous year and IMT (contrary to the case of vaginal infections) (Table 3). Regression analyses showed a significant association between total infections and sCVD, strengthened after adjusting for potential confounders. Women with the highest amount (2 or more) of total infections had 91% greater odds of sCVD (1.91; 95% CI 1.16,3.13; p-trend: 0.015), compared to women with no infections. A tendency could be appreciated between the amount of respiratory (1.19; 95% CI 0.82, 1.71; p-trend: 0.251) as well as urinary infections (1.29; 95% CI 0.86, 1.94; p-trend: 0.144) and sCVD, although not statistically significant (but not with vaginal infections) (Table 4).

**Table 3:** Adjusted differences, in percentage points (95% confidence intervals), in mean carotid IMT in 1946 women of the MTC according to categories of infectious events.

	0	1	2 or more	p-trend
Total infections				
N	246	390	1310	
Model 1	Reference	0.38 (-1.63,2.42)	1.04 (-0.70,2.81)	0.177
Model 2	Reference	0.34 (-1.67,2.39)	1.03 (-0.71,2.81)	0.173
Model 3 ^a^	Reference	0.40 (-1.55,2.38)	1.04 (-0.65,2.75)	0.170
Infections	0	1	2 or more	
Respiratory				
N	428	586	932	
Model 1	Reference	0.10 (-1.46,1.69)	0.32 (-1.13,1.79)	0.646
Model 2	Reference	0.08 (-1.49,1.68)	0.29 (-1.16,1.76)	0.767
Model 3 ^a^	Reference	0.13 (-1.39,1.67)	0.24 (-1.16,1.66)	0.736
Urinary				
N	1263	403	280	
Model 1	Reference	0.42 (-1.00,1.85)	1.31 (-0.34,2.98)	0.123
Model 2	Reference	0.42 (-1.00,1.86)	1.39 (-0.27,3.07)	0.105
Model 3 ^a^	Reference	0.36 (-1.01,1.75)	1.04 (-0.56,2.67)	0.201
Vaginal				
N	1252	364	330	
Model 1	Reference	-0.11 (-1.58,1.39)	-0.42 (-1.96,1.16)	0.614
Model 2	Reference	-0.13 (-1.61,1.38)	-0.34 (-1.89,1.24)	0.669
Model 3 ^a^	Reference	0.08 (-1.35,1.54)	-0.40 (-1.90,1.12)	0.668

Notes

Model 1: Adjusted for age and site

Model 2: Model 1 adjusted for socioeconomic status, education level, smoking, and alcohol intake

Model 3: Model 2 adjusted for diabetes, hypertension, hypercholesterolemia, BMI, and menopausal status

^a^ Three participants were excluded from Model 3 because they had a missing BMI

**Table 4:** Adjusted OR (95% CI) for sCVD in 1946 women of the MTC according to categories of infectious events.

	0	1	2 or more	p-trend
Total infections				
N	246	390	1310	
Model 1	Reference	1.40 (0.82,2.40)	1.72 (1.07,2.76)	0.021
Model 2	Reference	1.41 (0.82,2.42)	1.72 (1.07,2.77)	0.023
Model 3 ^a^	Reference	1.60 (0.91,2.80)	1.91 (1.16,3.13)	0.015
Infections	0	1	2 or more	
Respiratory				
N	428	586	932	
Model 1	Reference	0.85 (0.58,1.26)	1.10 (0.78,1.56)	0.413
Model 2	Reference	0.85 (0.58,1.27)	1.10 (0.77,1.56)	0.431
Model 3 ^a^	Reference	0.91 (0.61,1.37)	1.19 (0.82,1.71)	0.251
Urinary				
n	1263	403	280	
Model 1	Reference	1.28 (0.91,1.79)	1.36 (0.92,2.02)	0.067
Model 2	Reference	1.27 (0.91,1.78)	1.38 (0.93,2.04)	0.064
Model 3 ^a^	Reference	1.23 (0.87,1.75)	1.29 (0.86,1.94)	0.144
Vaginal				
n	1252	364	330	
Model 1	Reference	1.22 (0.85,1.74)	0.96 (0.64,1.45)	0.855
Model 2	Reference	1.22 (0.85,1.74)	0.95 (0.63,1.44)	0.889
Model 3 ^a^	Reference	1.26 (0.87,1.83)	0.91 (0.59,1.40)	0.989

Notes

Model 1: Adjusted for age and site.

Model 2: Model 1 adjusted for socioeconomic status, education level, smoking, and alcohol intake.

Model 3: Model 2 adjusted for diabetes, hypertension, hypercholesterolemia, BMI, and menopausal status.

^a^ Three participants were excluded from Model 3 because they had a missing BMI.

Stratified linear regression analyses showed that women aged ≥49 (study population median age) had a greater mean carotid IMT as they reported more infections during the last year (p-trend: 0.004). This was not observed among women aged <49 or stratified by median BMI (S1 Table). In the stratified logistic regression, women aged ≥49 and those with BMI ≥28.5 showed higher OR of sCVD as number of infections increased, but this was only statistically significant in women aged ≥49. Women aged <49 or with BMI <28.5 did not show a clear tendency (S2 Table).

Sensitivity analyses with different categorizations of total infectious events also showed significant associations between total infections and sCVD. Women with more than 3 infectious events during the previous year had greater odds of sCVD (OR 1.94; 95% CI 1.16, 3.23; p-trend: 0.027) than women with no infections. Those with an average of one infection per month, i.e. 12 or more events, showed much higher odds of sCVD (OR 3.02; 95% CI 1.23, 7.40; p-trend: 0.018). Linear regression analyses were non-significant, and a less strict definition of sCVD was not associated with total infectious events (S3–S7 Tables).

## Discussion

Our study shows an (to the best of our knowledge previously unreported) association between the amount of common non-serious infections (respiratory, urinary, or vaginal) among working women and high-resolution ultrasound-diagnosed atherosclerosis of the carotid arteries. We have observed that young adult women with more frequent infections (2 or more per year) had 91% greater odds of sCVD compared to those with none of those infections during their previous year of life.

Although some studies have assessed the relation between pathogen-specific infections and coronary heart disease risk [3, 4], studies about the association of self-reported more common, and often neglected, recurrent organ system-specific infections and cardiovascular disease were nonexistent. Therefore, we considered a questionnaire to be the best tool to assess our exposure, based on the fact that the epidemiology of common recurrent infections is extremely difficult to study since the vast majority of events tend to resolve spontaneously and patients often do not seek formal medical care at all.

Moreover, as in many other countries, Mexico does not have a universal electronic medical record that could allow the evaluation of the incidence of infectious events throughout participants’ lifespan. Since the Mexican Teachers’ Cohort is a multiethnic group scattered across the country in rural as well as urban areas, they may also have different types of access to both public and private health care that we may not be aware of.

Respiratory, urinary, and vaginal infections are common recurrent infections because they are the most frequent among adult women, and their symptoms could be identified and recalled by the participants [46–48]. Urinary tract infections were defined as events that required antibiotic treatment to help subjects distinguish between urinary and vaginal infections, since the latter frequently have a fungal etiology. Although respiratory infections may be mistaken with allergic manifestations, the latter are usually subacute or chronic, and seasonal; therefore, adults are familiar with their allergic symptoms and are able to distinguish between both diagnoses.

In contrast, gastrointestinal infections were excluded from the questionnaire since episodes of diarrhea (and abdominal pain) are highly frequent manifestations of common noninfectious diseases such as irritable bowel syndrome and lactose intolerance. Symptoms of these diseases could be hard to differentiate between infectious and non-infectious causes. For example, in everyday medical practice patients often erroneously attribute chronic or episodic diarrhea to bacterial o parasitic causes, demanding antibiotic therapy even despite negative microbiological laboratory results. Nevertheless, further research is needed to define the possible role of gastrointestinal infections in sCVD.

A strength of our study is the outcome assessment. Carotid IMT and carotid plaques have proven to be predictors of myocardial infarction and stroke and are used as a screening tool to asses cardiovascular risk [49]. Carotid arteries were examined by standardized neurologists blinded to the exposure using a state-of-the-art protocol. The high reproducibility of the IMT assessment was also established in two of the sites.

The amount of all infectious events during the previous year was directly associated with sCVD in this cross-sectional study, while site-specific infections were not. Respiratory and urinary infections, mainly viral and bacterial respectively, usually trigger a systemic inflammatory response [50, 51]. Specific viral and bacterial infectious agents, such as cytomegalovirus [23–25], influenza virus [29], Epstein-Barr virus [30] and *Chlamydia pneumoniae* [2], have been found to cause atherosclerosis through indirect mechanisms, such as increased circulating inflammatory factors, or increase acute and long-term risk of myocardial infarction and stroke. Although not statistically significant, we found a positive trend in the relation between both respiratory and urinary tract infections and sCVD, which is consistent with the proposed mechanism of chronic systemic inflammation. We believe this association could be not statistically significant due to insufficient sample size.

In contrast, vaginal infections did not show a trend or association with number of infections in the previous year. This could be explained because vaginal infections are predominantly fungal and superficial, generating local inflammatory responses that most likely do not translate in increased circulating inflammatory factors, one of the main proposed mechanisms of the effect of chronic infections in cardiovascular disease [52, 53].

Total infectious events during the previous year were associated with subclinical cardiovascular disease, association that was stronger among participants that reported an average of one infection per month. This is consistent with findings that aggregated activity of several infections is associated with atherosclerosis [35–38], even without other vascular risk factors [39]. Having found a relation with total infections and not with site-specific infections supports the hypothesis of the mayor role of low-grade chronic systemic (as opposed to local) inflammation in cardiovascular disease. As proposed by the pathogen burden theory, the total exposure to infections and chronic inflammation itself could have a role in the progression of atherosclerosis and increased cardiovascular risk rather than specific-site infections [4, 40–42].

Several studies have found that obesity induces production of pro-inflammatory molecules, promoting a low-level chronic inflammatory state [54–57], whose relation with aging has also been documented [58, 59]. To assess this potential interaction, we conducted stratified analyses using these factors. Although no statistically significant interaction was found, association seemed to be stronger among women with higher BMI (≥28.4 kg/m^2^) and age (≥49 years).

Somehow surprisingly, the prevalence of diabetes among the group that reported no infectious events during the previous year was higher. However, this difference almost disappeared when comparing only women with uncontrolled diabetes. An increasing gradient of poor metabolic control, the main immunocompromising factor in this population, can be observed as women reported more infections [60].

Our main explanatory line for the association we are establishing is chronic low-grade systemic inflammation, for which there are a series of biomarkers whose levels are known to be predictors of coronary events, the most widespread being high sensitivity CRP [2]. Acute infections are certainly capable of generating systemic inflammatory responses, but these could and should be transient, with biomarkers returning to normal levels in otherwise healthy subjects between events [16].

A potential limitation of our study is that no validated questionnaire exists to assess our particular exposure. Ours was designed by experienced clinicians considering culture and popular language, and one-year recall since this period is short enough to remember and long enough to be representative of adulthood exposure. The cross-sectional design of our study could also be questioned, nevertheless we registered the number of infections experienced during the previous year, while the measurement of the outcome was performed during clinical evaluation and therefore after the exposure. Another possible caveat could be that we did not review clinical records, however, as previously stated, people with these types of infections do not usually seek medical attention and therefore this information can only be collected asking participants to recall the previous year's infectious clinical pictures, a period that can be easily remembered. Finally, since we did not assess reported overall health, we were not able to adjust for this factor.

Although we do not have a previous measurement of IMT to compare with the one obtained during our clinical evaluation, we strengthened the assessment of our outcome through three different strategies. Firstly, we excluded participants who had a history of myocardial infarction or cerebrovascular disease. Secondly, we accurately measured early cardiovascular disease signs, such as IMT, by neurologists using standardized methods who were blinded to the exposure. Lastly, recruited participants were blinded to their outcome at the moment of answering the questionnaire registering the exposure.

Further research is needed to analyze this issue. If this kind of infectious burden is proven to be a risk factor for sCVD, public health policies could be directed to create awareness for prevention, timely diagnosis, and treatment of infections to avoid long term complications derived from sCVD.

## Supporting information

S1 Table Adjusted differences in carotid IMT stratified by age and BMI. Adjusted differences, in percentage points (95%CI), in mean carotid IMT in 1943^a^ women of the Mexican Teachers’ Cohort (MTC) according to categories of total infectious events, stratified by age and BMI medians, using Model 3.
(PDF)

S2 Table Adjusted OR for subclinical cardiovascular disease (sCVD) stratified by age and BMI. Adjusted OR (95%CI) for sCVD in 1943 ^a^ women of the MTC according to categories of total infectious events, stratified by age and BMI median, using Model 3.
(PDF)

S3 Table Adjusted differences in carotid IMT according to infectious events. Adjusted differences, in percentage points (95%CI), in mean carotid IMT in 1946 women of the MTC according to balanced categories of infectious events.
(PDF)

S4 Table Adjusted OR for sCVD according to infectious events. Adjusted OR (95%CI) for sCVD in 1946 women of the MTC according to balanced categories of infectious events.
(PDF)

S5 Table Adjusted differences in carotid IMT according to extreme categories of infectious events. Adjusted differences, in percentage points (95%CI), in mean carotid IMT in 1946 women of the MTC according to more extreme categories of infectious events.
(PDF)

S6 Table Adjusted OR for sCVD according to extreme categories of infectious events. Adjusted OR (95%CI) for sCVD in 1946 women of the MTC according to more extreme categories of infectious events.
(PDF)

S7 Table Adjusted OR for sCVD defined as right or left IMT ≥0.8 mm. Adjusted OR (95% CI) for sCVD in 1946 women of the MTC according to categories of total infectious events, with subclinical cardiovascular disease defined as right or left IMT ≥0.8 mm or plaque.
(PDF)

S1 File Infectious diseases questionnaire. Questionnaire applied during clinical evaluations in original language (Spanish) and English.
(PDF)
