# Supplementary material for: Association of recurrent common infections and subclinical cardiovascular disease in Mexican women
Source: PLoS One. 2021 Jan 26;16(1):e0246047. doi: 10.1371/journal.pone.0246047 (PMC7837493; doi:10.1371/journal.pone.0246047)
Supplement: S1 Table — Adjusted differences, in percentage points (95%CI), in mean carotid IMT in 1943a women of the Mexican Teachers’ Cohort (MTC) according to categories of total infectious events, stratified by age and BMI medians, using Model 3. (PDF) [file pone.0246047.s001.pdf]

S1 Table. Adjusted differences in carotid IMT stratified by age and BMI. Adjusted differences, in percentage points (95%CI), in mean carotid IMT in 1943[a] women of the Mexican Teachers' Cohort (MTC) according to categories of total infectious events, stratified by age and BMI medians, using Model 3.

	0	1	2 or more	p-trend	p-interaction
n	78	142	639	0.076	0.857
Age <49	Reference	-1.46 (-4.44,1.61)	-2.3 (-4.82,0.28)		
n	168	248	671	0.004	
Age ≥49	Reference	0.88 (-1.69,3.52)	2.87 (0.59,5.19)		
n	126	193	652	0.679	0.608
BMI <28.5	Reference	-0.2 (-2.87,2.55)	0.27 (-2.03,2.62)		
n	120	196	656	0.117	
BMI ≥28.5	Reference	0.91 (-1.92,3.82)	1.84 (-0.63,4.38)		

Notes

Model 3 was adjusted for age, site, socioeconomic status, education level, smoking, alcohol intake, diabetes, hypertension, hypercholesterolemia, BMI, and menopausal status.

[a] Three participants were excluded from Model 3 because they had a missing BMI.
