# Supplementary material for: Association of recurrent common infections and subclinical cardiovascular disease in Mexican women
Source: PLoS One. 2021 Jan 26;16(1):e0246047. doi: 10.1371/journal.pone.0246047 (PMC7837493; doi:10.1371/journal.pone.0246047)
Supplement: S2 Table — Adjusted OR (95%CI) for sCVD in 1943 a women of the MTC according to categories of total infectious events, stratified by age and BMI median, using Model 3. (PDF) [file pone.0246047.s002.pdf]

S2 Table. Adjusted OR for subclinical cardiovascular disease (sCVD) stratified by age and BMI. Adjusted OR (95%CI) for sCVD in 1943[a] women of the MTC according to categories of total infectious events, stratified by age and BMI median, using Model 3.

	0	1	2	p-trend	p-interaction
n	78	142	639	0.793	0.332
Age <49	Reference	1.17 (0.35,3.92)	1.19 (0.42,3.43)		
n	168	248	671	0.007	
Age ≥49	Reference	1.63 (0.86,3.08)	2.15 (1.23,3.75)		
n	126	193	652	0.041	0.895
BMI <28.5	Reference	1.06 (0.4,2.77)	1.85 (0.83,4.14)		
n	120	196	656	0.125	
BMI ≥28.5	Reference	2.05 (1,4.21)	2.02 (1.06,3.86)		

Notes

Model 3 was adjusted for age, site, socioeconomic status, education level, smoking, alcohol intake, diabetes, hypertension, hypercholesterolemia, BMI, and menopausal status.

[a] Three participants were excluded from Model 3 because they had a missing BMI.

S3 Table. Adjusted differences, in percentage points (95%CI), in mean carotid IMT in 1946 women of the MTC according to balanced categories of infectious events.

	No events (n=246)	1 event (n=390)	2 events (n=415)	3 events or more (n=895)	p - trend
Model 1	Reference	0.38 (-1.63,2.42)	1.05 (-0.95,3.09)	1.03 (-0.77,2.87)	0.25
Model 2	Reference	0.34 (-1.67,2.39)	1.03 (-0.97,3.08)	1.04 (-0.77,2.88)	0.239
Model 3[a]	Reference	0.40 (-1.54,2.38)	1.28 (-0.66,3.26)	0.92 (-0.83,2.70)	0.352

Notes

Model 1: Adjusted for age and site

Model 2: Model 1 adjusted for socioeconomic status, educational level, smoking, and alcohol intake

Model 3: Model 2 adjusted for diabetes, hypertension, hypercholesterolemia, BMI, and menopausal status

[a] Three participants were excluded from Model 3 because they had a missing BMI.

S4 Table. Adjusted OR (95%CI) for sCVD in 1946 women of the MTC according to balanced categories of infectious events.

	No events (n=246)	1 event (n=390)	2 events (n=415)	3 events or more (n=895)	p - trend
Model 1	Reference	1.40 (0.82,2.40)	1.60 (0.94,2.73)	1.78 (1.09,2.90)	0.023
Model 2	Reference	1.41 (0.82,2.42)	1.62 (0.95,2.76)	1.77 (1.09,2.89)	0.026
Model 3[a]	Reference	1.60 (0.91,2.80)	1.85 (1.06,3.22)	1.94 (1.16,3.23)	0.027

Notes

Model 1: Adjusted for age and site

Model 2: Model 1 adjusted for socioeconomic status, educational level, smoking, and alcohol intake

Model 3: Model 2 adjusted for diabetes, hypertension, hypercholesterolemia, BMI, and menopausal status

[a] Three participants were excluded from Model 3 because they had a missing BMI.

S5 Table. Adjusted differences, in percentage points (95%CI), in mean carotid IMT in 1946 women of the MTC according to more extreme categories of infectious events.

	No events (n=246)	1 event (n=390)	2 events (n=415)	3 to 11 events (n=841)	12 events or more (n=54)	p - trend
Model 1	Reference	0.38 (-1.63,2.42)	1.05 (-0.95,3.09)	1.04 (-0.78,2.89)	0.98 (-2.72,4.82)	0.445
Model 2	Reference	0.34 (-1.67,2.39)	1.03 (-0.97,3.08)	1.04 (-0.78,2.90)	0.90 (-2.81,4.75)	0.454
Model 3[a]	Reference	0.40 (-1.55,2.38)	1.28 (-0.66,3.26)	0.96 (-0.81,2.75)	0.33 (-3.23,4.02)	0.723

Notes

Model 1: Adjusted for age and site

Model 2: Model 1 adjusted for socioeconomic status, education level, smoking, and alcohol intake

Model 3: Model 2 adjusted for diabetes, hypertension, hypercholesterolemia, BMI, and menopausal status

[a] Three participants were excluded from Model 3 because they had a missing BMI

S6 Table. Adjusted OR (95%CI) for sCVD in 1946 women of the MTC according to more extreme categories of infectious events.

	No events (n=246)	1 event (n=390)	2 events (n=415)	3 to 11 events (n=841)	12 events or more (n=54)	p - trend
Model 1	Reference	1.40 (0.82,2.40)	1.60 (0.94,2.74)	1.73 (1.06,2.82)	2.83 (1.20,6.67)	0.012
Model 2	Reference	1.41 (0.82,2.42)	1.62 (0.95,2.76)	1.72 (1.05,2.81)	2.88 (1.22,6.81)	0.012
Model 3 [a]	Reference	1.60 (0.91,2.81)	1.85 (1.06,3.23)	1.88 (1.12,3.15)	3.02 (1.23,7.40)	0.018

Notes

Model 1: Adjusted for age and site

Model 2: Model 1 adjusted for socioeconomic status, education level, smoking, and alcohol intake

Model 3: Model 2 adjusted for diabetes, hypertension, hypercholesterolemia, BMI, and menopausal status

[a] Three participants were excluded from Model 3 because they had a missing BMI.

S7 Table. Adjusted OR (95% CI) for sCVD in 1946 women of the MTC according to categories of total infectious events, with subclinical cardiovascular disease defined as right or left IMT ≥0.8 mm or plaque.

	0	1	2 or more	p-trend
Total infections				
n	246	390	1310	
Model 1	Reference	1.18 (0.79,1.75)	1.20 (0.85,1.7)	0.630
Model 2	Reference	1.17 (0.79,1.74)	1.20 (0.85,1.7)	0.597
Model 3 [a]	Reference	1.24 (0.82,1.86)	1.24 (0.87,1.78)	0.673

Notes

Model 1: Adjusted for age and site

Model 2: Model 1 adjusted for socioeconomic status, education level, smoking, and alcohol intake

Model 3: Model 2 adjusted for diabetes, hypertension, hypercholesterolemia, BMI, and menopausal status

[a] Three participants were excluded from Model 3 because they had a missing BMI.

S8 Infectious diseases questionnaire. Questionnaire applied during clinical evaluations in original language (Spanish) and English.

Original language (Spanish)

En los últimos 12 meses…

¿… cuántas infecciones respiratorias (anginas, catarros, gripas, gripes, infecciones de garganta, resfriados, sinusitis, etc.) ha padecido?

Número de infecciones |___||___|

¿… cuántas infecciones urinarias (ardor al orinar, dolor en la vejiga, urgencia para orinar) ha padecido que hayan requerido antibiótico?

Número de infecciones |___||___|

¿… cuántas infecciones vaginales (ardor vaginal, comezón, flujo anormal) ha padecido?

Número de infecciones |___||___|

English

In the last 12 months…

How many respiratory infections (sore throat, colds, flu, throat infections, colds, sinusitis, etc.) have you had?

Number of infections | ___ || ___ |

How many urinary infections (burning when urinating, bladder pain, urge to urinate) that required antibiotics have you had?

Number of infections | ___ || ___ |

How many vaginal infections (vaginal burning, itching, abnormal discharge) have you had?

Number of infections | ___ || ___ |
