# Supplementary material for: Association of recurrent common infections and subclinical cardiovascular disease in Mexican women
Source: PLoS One. 2021 Jan 26;16(1):e0246047. doi: 10.1371/journal.pone.0246047 (PMC7837493; doi:10.1371/journal.pone.0246047)
Supplement: S3 Table — Adjusted differences, in percentage points (95%CI), in mean carotid IMT in 1946 women of the MTC according to balanced categories of infectious events. (PDF) [file pone.0246047.s003.pdf]

S3 Table. Adjusted differences in carotid IMT according to infectious events. Adjusted differences, in percentage points (95%CI), in mean carotid IMT in 1946 women of the MTC according to balanced categories of infectious events.

	No events (n=246)	1 event (n=390)	2 events (n=415)	3 events or more (n=895)	p - trend
Model 1	Reference	0.38 (-1.63,2.42)	1.05 (-0.95,3.09)	1.03 (-0.77,2.87)	0.25
Model 2	Reference	0.34 (-1.67,2.39)	1.03 (-0.97,3.08)	1.04 (-0.77,2.88)	0.239
Model 3[a]	Reference	0.40 (-1.54,2.38)	1.28 (-0.66,3.26)	0.92 (-0.83,2.70)	0.352

Notes

Model 1: Adjusted for age and site

Model 2: Model 1 adjusted for socioeconomic status, educational level, smoking, and alcohol intake

Model 3: Model 2 adjusted for diabetes, hypertension, hypercholesterolemia, BMI, and menopausal status

[a] Three participants were excluded from Model 3 because they had a missing BMI.
