# Supplementary material for: Association of recurrent common infections and subclinical cardiovascular disease in Mexican women
Source: PLoS One. 2021 Jan 26;16(1):e0246047. doi: 10.1371/journal.pone.0246047 (PMC7837493; doi:10.1371/journal.pone.0246047)
Supplement: S4 Table — Adjusted OR (95%CI) for sCVD in 1946 women of the MTC according to balanced categories of infectious events. (PDF) [file pone.0246047.s004.pdf]

S4 Table. Adjusted OR for sCVD according to infectious events. Adjusted OR (95%CI) for sCVD in 1946 women of the MTC according to balanced categories of infectious events.

	No events (n=246)	1 event (n=390)	2 events (n=415)	3 events or more (n=895)	p - trend
Model 1	Reference	1.40 (0.82,2.40)	1.60 (0.94,2.73)	1.78 (1.09,2.90)	0.023
Model 2	Reference	1.41 (0.82,2.42)	1.62 (0.95,2.76)	1.77 (1.09,2.89)	0.026
Model 3[a]	Reference	1.60 (0.91,2.80)	1.85 (1.06,3.22)	1.94 (1.16,3.23)	0.027

Notes

Model 1: Adjusted for age and site

Model 2: Model 1 adjusted for socioeconomic status, educational level, smoking, and alcohol intake

Model 3: Model 2 adjusted for diabetes, hypertension, hypercholesterolemia, BMI, and menopausal status

[a] Three participants were excluded from Model 3 because they had a missing BMI.
