# Supplementary material for: Association of recurrent common infections and subclinical cardiovascular disease in Mexican women
Source: PLoS One. 2021 Jan 26;16(1):e0246047. doi: 10.1371/journal.pone.0246047 (PMC7837493; doi:10.1371/journal.pone.0246047)
Supplement: S5 Table — Adjusted differences, in percentage points (95%CI), in mean carotid IMT in 1946 women of the MTC according to more extreme categories of infectious events. (PDF) [file pone.0246047.s005.pdf]

**S5 Table. Adjusted differences in carotid IMT according to extreme categories of infectious events.** Adjusted differences, in percentage points (95%CI), in mean carotid IMT in 1946 women of the MTC according to more extreme categories of infectious events.

| | No events (n=246) | 1 event (n=390) | 2 events (n=415) | 3 to 11 events (n=841) | 12 events or more (n=54) | p - trend |
|---|---|---|---|---|---|---|
| Model 1 | Reference | 0.38 (-1.63,2.42) | 1.05 (-0.95,3.09) | 1.04 (-0.78,2.89) | 0.98 (-2.72,4.82) | 0.445 |
| Model 2 | Reference | 0.34 (-1.67,2.39) | 1.03 (-0.97,3.08) | 1.04 (-0.78,2.90) | 0.90 (-2.81,4.75) | 0.454 |
| Model 3[a] | Reference | 0.40 (-1.55,2.38) | 1.28 (-0.66,3.26) | 0.96 (-0.81,2.75) | 0.33 (-3.23,4.02) | 0.723 |

**Notes**

Model 1: Adjusted for age and site

Model 2: Model 1 adjusted for socioeconomic status, education level, smoking, and alcohol intake

Model 3: Model 2 adjusted for diabetes, hypertension, hypercholesterolemia, BMI, and menopausal status

[a] Three participants were excluded from Model 3 because they had a missing BMI
