# Supplementary material for: Association of recurrent common infections and subclinical cardiovascular disease in Mexican women
Source: PLoS One. 2021 Jan 26;16(1):e0246047. doi: 10.1371/journal.pone.0246047 (PMC7837493; doi:10.1371/journal.pone.0246047)
Supplement: S6 Table — Adjusted OR (95%CI) for sCVD in 1946 women of the MTC according to more extreme categories of infectious events. (PDF) [file pone.0246047.s006.pdf]

S6 Table. Adjusted OR for sCVD according to extreme categories of infectious events. Adjusted OR (95%CI) for sCVD in 1946 women of the MTC according to more extreme categories of infectious events.

	No events (n=246)	1 event (n=390)	2 events (n=415)	3 to 11 events (n=841)	12 events or more (n=54)	p - trend
Model 1	Reference	1.40 (0.82,2.40)	1.60 (0.94,2.74)	1.73 (1.06,2.82)	2.83 (1.20,6.67)	0.012
Model 2	Reference	1.41 (0.82,2.42)	1.62 (0.95,2.76)	1.72 (1.05,2.81)	2.88 (1.22,6.81)	0.012
Model 3 [a]	Reference	1.60 (0.91,2.81)	1.85 (1.06,3.23)	1.88 (1.12,3.15)	3.02 (1.23,7.40)	0.018

Notes

Model 1: Adjusted for age and site

Model 2: Model 1 adjusted for socioeconomic status, education level, smoking, and alcohol intake

Model 3: Model 2 adjusted for diabetes, hypertension, hypercholesterolemia, BMI, and menopausal status

[a] Three participants were excluded from Model 3 because they had a missing BMI.
