# Supplementary material for: Association of recurrent common infections and subclinical cardiovascular disease in Mexican women
Source: PLoS One. 2021 Jan 26;16(1):e0246047. doi: 10.1371/journal.pone.0246047 (PMC7837493; doi:10.1371/journal.pone.0246047)
Supplement: S7 Table — Adjusted OR (95% CI) for sCVD in 1946 women of the MTC according to categories of total infectious events, with subclinical cardiovascular disease defined as right or left IMT ≥0.8 mm or plaque. (PDF) [file pone.0246047.s007.pdf]

S7 Table. Adjusted OR for sCVD defined as right or left IMT ≥0.8 mm. Adjusted OR (95% CI) for sCVD in 1946 women of the MTC according to categories of total infectious events, with subclinical cardiovascular disease defined as right or left IMT ≥0.8 mm or plaque.

	0	1	2 or more	p-trend
Total infections				
n	246	390	1310	
Model 1	Reference	1.18 (0.79,1.75)	1.20 (0.85,1.7)	0.630
Model 2	Reference	1.17 (0.79,1.74)	1.20 (0.85,1.7)	0.597
Model 3 [a]	Reference	1.24 (0.82,1.86)	1.24 (0.87,1.78)	0.673

Notes

Model 1: Adjusted for age and site

Model 2: Model 1 adjusted for socioeconomic status, education level, smoking, and alcohol intake

Model 3: Model 2 adjusted for diabetes, hypertension, hypercholesterolemia, BMI, and menopausal status

[a] Three participants were excluded from Model 3 because they had a missing BMI.
