# Supplementary material for: Association of recurrent common infections and subclinical cardiovascular disease in Mexican women
Source: PLoS One. 2021 Jan 26;16(1):e0246047. doi: 10.1371/journal.pone.0246047 (PMC7837493; doi:10.1371/journal.pone.0246047)
Supplement: S1 File — Questionnaire applied during clinical evaluations in original language (Spanish) and English. (PDF) [file pone.0246047.s008.pdf]

**S1 File. Infectious diseases questionnaire.** Questionnaire applied during clinical evaluations in original language (Spanish) and English.

## Original language (Spanish)

En los últimos 12 meses…

¿… cuántas infecciones respiratorias (anginas, catarros, gripas, gripes, infecciones de garganta, resfriados, sinusitis, etc.) ha padecido?

Número de infecciones |___||___|

¿… cuántas infecciones urinarias (ardor al orinar, dolor en la vejiga, urgencia para orinar) ha padecido que hayan requerido antibiótico?

Número de infecciones |___||___|

¿… cuántas infecciones vaginales (ardor vaginal, comezón, flujo anormal) ha padecido?

Número de infecciones |___||___|

## English

In the last 12 months…

How many respiratory infections (sore throat, colds, flu, throat infections, colds, sinusitis, etc.) have you had?

Number of infections | ___ || ___ |

How many urinary infections (burning when urinating, bladder pain, urge to urinate) that required antibiotics have you had?

Number of infections | ___ || ___ |

How many vaginal infections (vaginal burning, itching, abnormal discharge) have you had?

Number of infections | ___ || ___ |
